# A Transcriptomic Severity Classifier IMX-SEV-3b to Predict Mortality in Intensive Care Unit Patients with COVID-19: A Prospective Observational Pilot Study

**DOI:** 10.3390/jcm12196197

**Published:** 2023-09-26

**Authors:** Katrijn Daenen, Kirby Tong-Minh, Oliver Liesenfeld, Sara C. M. Stoof, Jilske A. Huijben, Virgil A. S. H. Dalm, Diederik Gommers, Eric C. M. van Gorp, Henrik Endeman

**Affiliations:** 1 Department of Intensive Care, Erasmus University Medical Center, 3015 GD Rotterdam, The Netherlandsj.a.huijben@erasmusmc.nl (J.A.H.); d.gommers@erasmusmc.nl (D.G.); h.endeman@erasmusmc.nl (H.E.); 2 Department of Viroscience, Erasmus University Medical Center, 3015 GD Rotterdam, The Netherlandse.vangorp@erasmusmc.nl (E.C.M.v.G.); 3 Inflammatix Inc., Sunnyvale, CA 94085, USA; 4 Department of Immunology, Erasmus University Medical Center Rotterdam, 3015 GD Rotterdam, The Netherlands; v.dalm@erasmusmc.nl; 5 Department of Internal Medicine, Division of Allergy & Clinical Immunology, Erasmus University Medical Center, 3015 GD Rotterdam, The Netherlands

**Keywords:** COVID-19, infectious diseases, virology, severity, transcriptome, classifier, prediction

## Abstract

The prediction of disease outcomes in COVID-19 patients in the ICU is of critical importance, and the examination of host gene expressions is a promising tool. The 29-host mRNA Inflam-matix-Severity-3b (IMX-SEV-3b) classifier has been reported to predict mortality in emergency department COVID-19 patients and surgical ICU patients. The accuracy of the IMX-SEV-3b in predicting mortality in COVID-19 patients admitted to the ICU is yet unknown. Our aim was to investigate the accuracy of the IMX-SEV-3b in predicting the ICU mortality of COVID-19 patients. In addition, we assessed the predictive performance of routinely measured biomarkers and the Sequential Organ Failure Assessment (SOFA) score as well. This was a prospective observational study enrolling COVID-19 patients who received mechanical ventilation on the ICU of the Erasmus MC, the Netherlands. The IMX-SEV-3b scores were generated by amplifying 29 host response genes from blood collected in PAXgene^®^ Blood RNA tubes. A severity score was provided, ranging from 0 to 1 for increasing disease severity. The primary outcome was the accuracy of the IMX-SEV-3b in predicting ICU mortality, and we calculated the AUROC of the IMX-SEV-3b score, the biomarkers C-reactive protein (CRP), D-dimer, ferritin, leukocyte count, interleukin-6 (IL-6), lactate dehydrogenase (LDH), neutrophil-to-lymphocyte ratio (NLR), procalcitonin (PCT) and the SOFA score. A total of 53 patients were included between 1 March and 30 April 2020, with 47 of them being included within 72 h of their admission to the ICU. Of these, 18 (34%) patients died during their ICU stay, and the IMX-SEV-3b scores were significantly higher in non-survivors compared to survivors (0.65 versus 0.57, *p* = 0.05). The Area Under the Receiver Operating Characteristic Curve (AUROC) for prediction of ICU mortality by the IMX-SEV-3b was 0.65 (0.48–0.82). The AUROCs of the biomarkers ranged from 0.52 to 0.66, and the SOFA score had an AUROC of 0.81 (0.69–0.93). The AUROC of the pooled biomarkers CRP, D-dimer, ferritin, leukocyte count, IL-6, LDH, NLR and PCT for prediction of ICU mortality was 0.81 (IQR 0.69–0.93). Further validation in a larger interventional trial of a point-of-care version of the IMX-SEV-3b classifier is warranted to determine its value for patient management.

## 1. Introduction

The emergence of the SARS-CoV-2 virus caused the global Coronavirus disease 2019 (COVID-19) pandemic and had a major impact on healthcare systems worldwide [1,2]. However, substantial variation exists in the clinical course of the disease between patients, ranging from asymptomatic infection to life-threatening Acute Respiratory Distress Syndrome (ARDS) [3,4]. It is presumed that a disparity in the level of inflammation between patients contributes to this heterogeneous clinical course [5,6,7]. Patients experiencing progressive respiratory failure frequently fulfill the Berlin criteria for moderate and severe ARDS and require admission to the intensive care unit (ICU) [8,9]. These patients often undergo prolonged ICU stays and have high mortality rates [10,11].

The identification of patients at risk of a severe outcome is of great importance, as clinicians may be able to act on signs of clinical deterioration at an earlier stage with intensified monitoring and targeted therapies. Furthermore, it enables clinicians to provide more precise information to patients and their relatives about the prognosis of the disease. Previous research has mainly focused on protein-based biomarkers for COVID-19 prognostication [12,13,14]. Several studies have found that elevated levels of biomarkers of inflammation and coagulation, including procalcitonin (PCT), C-reactive protein (CRP), Interleukin-6 (IL-6), D-dimer and lactate dehydrogenase (LDH) are associated with a severe outcome [15,16,17,18,19]. Also, several risk scores, mainly based on vital signs and laboratory parameters, have been studied to predict outcomes in COVID-19, such as the Sequential Organ Failure Assessment (SOFA) score, the Acute Physiology and Chronic Health Evaluation IV (APACHE-IV) score and the 4C (Coronavirus Clinical Characterisation Consortium) Mortality Score [20,21,22,23,24]. However, the potential for timely intervention may be lost when vital signs have already worsened and protein-based biomarkers are elevated. Furthermore, the prognostic accuracy of single protein-based serum biomarkers in predicting mortality is often insufficient [14,25], therefore supporting the need for the exploration of alternative tools involving underlying pathways of the immune system.

A relatively novel approach for predicting disease severity is the examination of host gene expression by transcriptome analyses. During infection, various genes are upregulated and downregulated in response to the presence of pathogens and immune system activation [26]. The measurement of gene expression levels provides valuable insights into the complex molecular mechanisms underlying the immune response to infection. Gene expression profiles associated with poor outcomes have been identified in various contexts and diseases, including patients with sepsis [27,28]. The Inflammatix Severity-3b (IMX-SEV-3b) classifier quantifies 29-mRNA host response genes from peripheral blood, which encompasses genes associated with diverse aspects of the immune response, such as the interferon and antigen presentation pathways [29]. For the initial selection of the 29 mRNAs databases, heterogeneous cohorts from diverse geographies, age groups, ethnicities, diagnoses, and settings of care were used, including inpatient wards, emergency departments, outpatient departments, and intensive care units. This classifier has proven to be a reliable predictor of severe outcomes in COVID-19 patients in the emergency department [30]. Furthermore, the IMX-SEV-3b successfully predicted 30-day mortality in surgical ICU patients with sepsis or suspected sepsis [31]. Whether IMX-SEV-3b will be able to predict mortality in COVID-19 ARDS patients in the ICU has not yet been studied. Therefore, the goal of this study is to investigate the accuracy of the IMX-SEV-3b classifier in predicting mortality in COVID-19 patients admitted to the ICU.

## 2. Materials and Methods

### 2.1. Study Design

This study was a prospective single-center cohort study conducted in the Netherlands. We included patients admitted to the ICU of Erasmus University Medical Center in Rotterdam with a confirmed COVID-19 infection between 1 March 2020 and 30 April 2020. The study was approved by the local Medical Ethics Review Committee under protocol number MEC-2017-417 and conducted according to the principles of the World Medical Association Declaration of Helsinki.

Eligibility for inclusion in this study was defined as follows:

### 2.2. Inclusion and Exclusion Criteria

- Patients who were at least 18 years of age.
- Patients who were admitted to the ICU with a Polymerase Chain Reaction (PCR)-confirmed COVID-19 infection.
- Patients or their legal representatives gave informed consent for inclusion and PAXgene Blood RNA whole blood draw, as well as analysis and shipment of their sample to Inflammatix Inc., Sunnyvale, CA, USA.

There were no exclusion criteria.

### 2.3. Data Collection

Patient data, including demographics, body mass index and comorbidities, were collected at baseline. Biomarker levels, including CRP, D-dimer, ferritin, leukocyte count, IL-6, LDH, NLR and PCT, were evaluated and collected on the day of inclusion as close to 6 AM as feasible. The APACHE-IV score was recorded from the electronic health record on the day of ICU admission and the SOFA score was recorded daily. Patients were monitored until their discharge from the ICU or until their death in the ICU.

### 2.4. Primary Outcome

The primary outcome of this study was the accuracy of the IMX-SEV-3b classifier in predicting mortality during the ICU stay, expressed as Area Under the Receiver Operating Characteristic Curve (AUROC). We compared this to the accuracy of the biomarkers CRP, D-dimer, ferritin, leukocyte count, IL-6, LDH, NLR and PCT, and the SOFA score, which were all evaluated on the day of inclusion, in predicting ICU mortality.

### 2.5. PAXgene Collection and Amplification of Target Genes

Whole blood (2.5 mL) was collected in PAXgene Blood RNA tubes (PreAnalytics, Hombrechtikon, Switzerland) on the day of inclusion, and subsequently aliquoted and stored at −80 °C until analysis. To perform target gene amplification and generate IMX-SEV-3b classifier results, the samples were shipped on dry ice to the Inflammatix laboratory (Burlingame, CA, USA) in batches, following the procedure previously described [29]. RNA was extracted from PAXgene tubes using the RNeasy^®^ Plus Micro Kit (QIAGEN, Hilden, Germany) on the QiaCube^®^ Connect instrument. The NanoString nCounter^®^ SPRINT profiler (NanoString, Seattle, WA, USA) was then used to quantify the 29 host target mRNAs, as well as four housekeeping genes for normalization (*CDIPT*, *KPNA6*, *RREB1* and *YWHAB*) from 150 ng of isolated RNA. RNA concentrations were determined for 29 genes of various aspects of the immune response and included *CEACAM1*, *ZDHHC19*, *C9orf95*, *GNA15*, *BATF*, *C3AR1*, *KIAA1370*, *TGFBI*, *MTCH1*, *RPGRIP1*, *HLA-DPB1*, *HK3*, *TNIP1*, *GPAA1*, *CTSB*, *IFI27*, *JUP*, *LAX1*, *DEFA4*, *CD163*, *RGS1*, *PER1*, *HIF1A*, *SEPP1*, *C11orf74*, *CIT*, *LY86*, *TST* and *KCNJ2* [29]. Operators at Inflammatix were blinded to all other study results, and the NanoString data were subjected to direct application of the SEV-3b classifier, blinded to clinical outcomes.

### 2.6. The IMX-SEV-3b Classifier

The machine learning algorithm IMX-SEV-3b reads and interprets the results of gene amplification. The results of the IMX-SEV-3b classifier were provided as absolute numerical scores between 0 and 1 for increasing severity prediction. To aid clinical usability, predefined thresholds categorized severity scores into five interpretation bands labeled ‘Very Low’, ’Low’, ’Moderate’, ‘High’ and ‘Very High’ illness severity. For each interpretation band, specificity and sensitivity were calculated to show accuracy.

### 2.7. Statistical Analysis

Continuous variables that were normally distributed are reported as a mean with standard deviation (SD), and non-normally distributed variables as a median with interquartile range (IQR). Categorical variables are shown as proportions. We compared differences between groups with a Chi-Square test and Student’s *t*-test or Mann–Whitney U test as appropriate. *p*-values of 0.05 or less were yielded statistically significant. For the primary analysis, we calculated the AUROC of the IMX-SEV-3b score and the biomarkers CRP, D-dimer, ferritin, leukocyte count, IL-6, LDH, NLR and PCT on the day of inclusion for ICU mortality. To test the performance of an important clinical score used in the ICU, we also calculated the AUROC of the SOFA score on the day of inclusion for ICU mortality. We used a multivariate logistic regression model combining the biomarkers in a composite biomarker model and calculated the AUROC of this logistic regression model. Finally, we calculated the sensitivity, specificity, and likelihood ratios for the 5 interpretation bands generated by the IMX-SEV-3b classifier. We used R (version 4.0.1) for statistical analysis.

## 3. Results

Between 1 March 2020 and 30 April 2020, a total of 152 patients were admitted to the ICU with a PCR-confirmed SARS-CoV-2 infection. Of these patients, 53 provided informed consent for the inclusion and collection of a PAXgene Blood RNA tube, and they all met the criteria outlined in the Berlin definition of ARDS [8]. A total of 80% of the patients were initially admitted to another hospital in the Netherlands and transferred to the Erasmus MC. The median time from the first day of hospital admission to ICU admission at Erasmus MC was 5 days. The majority of patients (27 out of 53) were included in our study within 24 h after ICU admission, and 47 out of 53 patients were included within 72 h after ICU admission. The median age was 66 years (IQR 60.0–72.0); 83% of patients were male, and the median BMI was 27.4 kg/m^2^ (IQR 25.3–30.2) (Table 1).

A total of 29 (54.7%) patients were diagnosed with a pulmonary embolism during their hospital stay, and the incidence was similar between non-survivors (61.1%) and survivors (51.4%) (*p* = 0.704). None of the patients had been subjected to corticosteroid therapy for COVID-19 indications prior to their admission to intensive care. In total, 18 patients (34%) died during their ICU stay. There were no significant differences in age, sex, BMI and comorbidity between survivors and non-survivors (Table 1). Similarly, the results of laboratory tests on the day of inclusion in the study, CRP, D-dimer, ferritin, leukocyte count, IL-6, LDH, NLR and PCT did not differ significantly between survivors and non-survivors (Table 2).

The mean IMX-SEV-3b was significantly lower (0.58) in survivors versus non-survivors (0.66) (*p* = 0.050). The AUROC of the IMX-SEV-3b for the prediction of ICU mortality was 0.65 (IQR 0.48–0.82). The calculated AUROCs of the biomarkers are shown in Table 3. The AUROC of the pooled biomarkers CRP, D-dimer, ferritin, leukocyte count, IL-6, LDH, NLR and PCT for the prediction of ICU mortality was 0.81 (IQR 0.69–0.93). The SOFA score had an AUROC of 0.81 (IQR 0.69–0.93) for the prediction of ICU mortality, and the score was significantly lower (6.00) in survivors compared to non-survivors (9.50).

When stratifying patients into the IMX-SEV-3b illness severity interpretation bands, 1 patient (2%) fell into the Very Low, 31 (58%) into the Low, 15 (28%) into the Moderate, 2 (4%) into the High and 4 (8%) into the Very High severity category (Table 4). All patients in the Very High illness severity band died in the ICU. The specificity of the ‘rule-in’ Very High, High and Moderate interpretation bands was, respectively, 100%, 94% and 74%, and the sensitivity of the ‘rule-out’ Very Low and Low interpretation bands was 100% and 56% (Table 4).

We performed a sub-analysis comparing AUROCs in the 21 patients who were enrolled within 5 days of hospital admission vs. those enrolled after 5 days of hospital admission (Table 5). Within the group of patients enrolled within 5 days the mortality rate was 28.6%, and within the group of patients enrolled after 5 days the mortality rate was 37.5%. The AUROCs of the IMX-SEV-3b and that of the biomarkers, CRP, D-dimer, IL-6, ferritin, leukocyte count and LDH are shown in Table 5.

## 4. Discussion

The clinical circumstances experienced during the first wave of the pandemic posed unique challenges due to the presence of the unfamiliar SARS-CoV-2 virus and the resulting capacity constraints. The high mortality rate highlighted the pressing need for improved tools that are able to predict which patients are at the highest risk of mortality in the ICU [32,33]. In our study, we investigated the accuracy of the host response classifier, IMX-SEV-3b, in predicting the ICU mortality of COVID-19 patients. Subsequently, we conducted a similar evaluation for several routinely measured biomarkers and the SOFA score. By achieving a non-significant AUROC for mortality prediction in this cohort, the IMX-SEV-3b classifier did perform below our initial expectations.

In relation to the existing literature on the IMX-SEV-3b classifier, the results of our study demonstrated lower performance than previously published. In comparison, Galtung et al. observed an AUROC of 0.84 for predicting in-hospital mortality in their cohort of patients presenting to the emergency department with suspected acute infections [30], and Brakenridge et al. observed an AUROC of 0.81 for predicting 30-day mortality in a surgical intensive care unit [31]. Given the fact that the IMX-SEV-3b has been scarcely tested within other ICU populations, it could be contemplated that the classifier’s performance might be more pronounced during an earlier stage of infection, such as when patients initially present at the emergency department.

However, since the performance of the other biomarkers in predicting mortality was also poor, one of the explanations might be related to the characteristics of the patient cohort in our study. Mainly due to capacity constraints in other hospitals, 80% of the patients were transferred from another hospital to the Erasmus MC, resulting in a median duration of 5 days from their initial hospital admission to study inclusion. Additionally, 51% of the patients were included within 24 h after ICU admission, with the day of inclusion varying among patients. It is presumed that the timing of ICU admission typically aligns with the point at which patients experience clinical deterioration necessitating invasive mechanical ventilation. A state of hyperinflammation present within both the pulmonary and circulatory systems plays a contributing role. Since the host immune response reduces over time, as illustrated by a decrease in cytokine levels and a quantitative reduction in specific mRNA levels, this may suggest that the optimal time window for the determination might have been missed for some of our patients. This could potentially have resulted in a lower predictive value of immune response-based tools, like the IMX-SEV-3b classifier and the biomarkers of inflammation. In this regard, we performed a sub-analysis comparing the AUROCs of patients who were enrolled within 5 days of hospital admission vs. those enrolled after 5 days of hospital admission. Although there seems to be a trend of improvement in the group of patients enrolled within 5 days supporting this hypothesis, it is noteworthy that the AUROC of the IMX-SEV-3b remains statistically insignificant within this subgroup. Likewise, the AUROCs of the biomarkers fail to attain statistical significance in this group, with the exception of LDH.

Corticosteroids, which are nowadays the cornerstone in the treatment of COVID-19, are also known to have an effect on inflammatory profiles. However, at the time of our study, guidelines had not yet incorporated corticosteroid treatment for COVID-19 management, so potential influence on the inflammatory profile is presumed to be absent. As corticosteroids were not included in treatment, this could raise the question of whether the results of our study can be extrapolated to COVID-19 patients treated with corticosteroids. Taking into account the fact that the cohorts used for the selection of the 29 mRNAs and the development of the IMX-SEV-3b classifier were diverse and included patients on immunosuppressive therapy, including corticosteroids, we expect little concern regarding extrapolation.

Many prior studies have indicated that integrating multiple biomarkers into a model enhances the predictive accuracy of ICU outcomes. The analysis of the composite biomarker model showed a substantial improvement, with a statistically significant AUROC. However, this model is highly at risk of overfitting due to the large number of predictors and a cohort with only 53 patients, and therefore, caution must be applied when interpreting this. The SOFA score showed a statistically significant AUROC for mortality prediction in this cohort. One possible reason might be that the SOFA score relies on parameters of organ failure rather than markers of inflammation, which could be less time sensitive in its predictive value. Although the SOFA score is the most well-known score in ICU practice, it is developed to sequentially evaluate the patient’s condition and is not able to provide instant individual predictions about mortality. In contrast, the illness severity interpretation bands provided by the IMX-SEV-3b classifier may allow for earlier clinical decision-making; the most actionable outer bands showed high sensitivity and specificity.

This study has several limitations. Firstly, the sample size is limited to 53 patients from a single center, and therefore, caution must be applied to the generalizability of the results. In light of the elevated risk of overfitting associated with the small sample size, we refrained from conducting statistical comparisons between the performance of the IMX-SEV-3b, biomarkers and SOFA score. Secondly, the inclusion of patients did not uniformly occur on the same day after admission, and their clinical trajectory before ICU admission varied highly. This contributed to the heterogeneity of the cohort. However, it is important to acknowledge that this was the prevailing clinical situation during the first wave of the COVID-19 pandemic that we encountered. Thirdly, the IMX-SEV-3b classifier is not feasible for clinical implementation yet, because the results are currently analyzed by the test manufacturer on a reference platform (NanoString). However, the upcoming availability of the point-of-care test directly from blood with a rapid turnaround time is expected to provide clinicians with immediate usable results.

The outcomes of our study underline the challenges of outcome prediction within a heterogeneous cohort of critically ill ICU patients. Moreover, these findings warrant further studies on the predictive value of the IMX-SEV-3b in the ICU. We acknowledge that the number of COVID-19 patients admitted to ICUs has significantly decreased. As the IMX-SEV-3b was validated using a 30-day all-cause in-hospital mortality endpoint [29], the classifier may be applied to other cohorts of critically ill patients with infectious diseases in the ICU. By incorporating repeated measurements of transcriptomic analyses in future studies, valuable insights into the prognostic value of transcriptome analyses over time may be provided, and critical windows for accurate prediction in the ICU may be identified [34].

## 5. Conclusions

In this observational pilot study, the mean IMX-SEV-3b score of the 29-gene host response classifier was significantly lower (0.58) in survivors compared to non-survivors (0.66). The AUROCs of the IMX-SEV-3b and the assessed biomarkers failed to achieve statistical significance in predicting mortality within this COVID-19 patient cohort, in contrast to the SOFA score, which did exhibit statistical significance. Further prospective studies are required to test the IMX-SEV-3b classifier in the ICU.

## Figures and Tables

**Table 1:** Baseline patient characteristics.

Patient Characteristics	All Patients	Survivors	Non-Survivors	*p*-Value
	n = 53	n = 35	n = 18	
Age, median [IQR]	66.0 (60.0–72.0)	65.0 (58.0–71.0)	69.0 (64.3–72.8)	0.14
Sex, male	44 (83.0%)	29 (82.9%)	15 (83.3%)	1
BMI, median [IQR]	27.4 (25.3–30.2)	27.5 (25.5–30.1)	26.3 (25.1–29.9)	0.98
Missing	1	1	0	
APACHE-IV (%) *	18.20 (11.10–30.20)	12.00 (9.00–19.10)	32.0 (24.40–50.80)	<0.001
Comorbidities Cardiovascular disease	6 (11.3%)	3 (8.6%)	3 (16.7%)	0.67
Pulmonary disease	6 (11.3%)	4 (11.4%)	2 (11.1%)	1
Neurological disease	2 (3.8%)	1 (2.9%)	1 (5.6%)	1
Renal disease	0 (100%)	0 (100%)	0 (100%)	1
Diabetes mellitus	11 (20.8%)	5 (14.3%)	6 (33.3%)	0.21
Immunodeficiency	4 (7.5%)	3 (8.6%)	1 (5.6%)	1
Autoimmune disease	4 (7.5%)	3 (8.6%)	1 (5.6%)	1

BMI: body mass index; APACHE: acute physiology, age, and chronic health evaluation. * Estimated Mortality Rate APACHE-IV Score.

**Table 2:** Biomarkers, SOFA and IMX-SEV-3b scores at inclusion.

Characteristics	All Patients	Survivors	Non-Survivors	*p*-Value
	n = 53	n = 35	n = 18	
CRP (mg/L)	277 (178–345)	304 (174–341)	210 (178–347)	0.74
D-dimer (mg/L)	2.48 (1.38–5.22)	2.42 (1.18–3.96)	3.86 (1.81–8.47)	0.17
Ferritin (mg/L)	1580 (974–2680)	1660 (1130–2730)	1300 (989–2240)	0.33
Leukocyte count (×10^9^/L)	8.74 (6.53–10.7)	8.31 (6.47–9.53)	10.30 (7.57–12.30)	0.06
IL-6 (pg/mL)	161 (88–307)	143 (91.5–246)	197 (87.3–395)	0.36
LDH (U/L)	322 (271–394)	320 (264–360)	341 (293–456)	0.06
NLR	7.45 (4.78–10.7)	6.72 (4.69–9.92)	8.85 (5.03–12.40)	0.22
PCT (ng/mL)	0.97 (0.470–2.70)	0.77 (0.36–2.04)	1.44 (0.90–3.20)	0.28
SOFA score	7.00 (6.00–10.00)	7.00 (6.00–8.00)	11.00 (9.00–11.00)	<0.001
IMX-SEV-3b score *	0.582 ± 0.13	0.575 ± 0.11	0.660 ± 0.15	0.050

CRP: C-reactive protein. LDH: lactate dehydrogenase. IL-6: interleukin-6. NLR: neutrophil-to-lymphocyte ratio. PCT: procalcitonin. SOFA: Sequential Organ Failure Assessment. * Mean ± SD; all others are reported as median (IQR).

**Table 3:** AUROCs of biomarkers, SOFA and IMX-SEV-3b.

Test/Biomarker/Clinical Score	AUROC (95% CI)
CRP (mg/L)	0.52 (0.34–0.70)
D-dimer (mg/L)	0.62 (0.45–0.79)
Ferritin (mg/L)	0.58 (0.42–0.75)
Leukocyte count (×109/L)	0.66 (0.48–0.83)
IL-6 (pg/mL)	0.58 (0.40–0.76)
LDH (U/L)	0.56 (0.37–0.74)
NLR	0.60 (0.43–0.78)
PCT (ng/mL)	0.59 (0.43–0.76)
IMX-SEV-3b	0.65 (0.48–0.82)
SOFA score	0.81 (0.69–0.93)
Pooled biomarker model	0.81 (0.69–0.93)

CRP: c-reactive protein. IL-6: interleukin-6. LDH: lactate dehydrogenase. NLR: neutrophil-to-lymphocyte ratio. PCT: procalcitonin. SOFA: Sequential Organ Failure Assessment. The pooled biomarker model included the biomarkers CRP, D-dimer, ferritin, leukocyte count, IL-6, LDH, NLR and PCT.

**Table 4:** Performance of the IMX-SEV-3b in predicting ICU mortality.

IMX-SEV-3b Severity Score		Survival Status		IMX-SEV-3b Performance per Band			
		Survivor	Non-Survivor	% Patients in Band	Sensitivity	Specificity	Likelihood Ratio
**IMX-SEV-3b category**	**Very High**	0	4	8%	22%	100%	Inf.
	**High**	2	0	4%	0%	94%	0.00
	**Moderate**	9	6	28%	33%	74%	1.30
	**Low**	23	8	58%	56%	66%	0.68
	**Very Low**	1	0	2%	100%	3%	0.00

Inf: infinite.

**Table 5:** Sub-analysis of AUROCs for biomarkers, IMX-SEV-3b and SOFA scores in patients included in the study within 5 days of hospital admission vs. those admitted after 5 or more days of hospital admission.

Test/Biomarker/Clinical Score	AUROC (95% CI) Patients Admitted within 5 Days (n = 21)	AUROC (95% Cl) Patients Admitted after 5 Days (n = 32)
CRP (mg/L)	0.59 (0.26–0.92)	0.60 (0.39–0.81)
D-dimer (mg/L)	0.72 (0.39–1.00)	0.56 (0.35–0.77)
Ferritin (mg/L)	0.60 (0.28–0.92)	0.59 (0.38–0.80)
Leukocyte count (×10^9^/L)	0.71 (0.44–0.97)	0.62 (0.38–0.86)
IL-6 (pg/mL)	0.67 (0.33–1.00)	0.55 (0.33–0.77)
LDH (U/L)	0.79 (0.56–1.00)	0.58 (0.37–0.80)
NLR	0.38 (0.10–0.66)	0.62 (0.40–0.84)
PCT (ng/mL)	0.56 (0.29–0.84)	0.63 (0.41–0.84)
IMX-SEV-3b	0.72 (0.43–1.00)	0.60 (0.37–0.83)
SOFA score	0.74 (0.49–1.00)	0.86 (0.73–0.99)

## Data Availability

The datasets used and/or analyzed during the current study are available from the corresponding author on reasonable request.
